# Melanopsin-Driven Pupil Response and Light Exposure in Non-seasonal Major Depressive Disorder

**DOI:** 10.3389/fneur.2018.00764

**Published:** 2018-09-13

**Authors:** Beatrix Feigl, Govinda Ojha, Leanne Hides, Andrew J. Zele

**Affiliations:** ^1^Medical Retina and Visual Science Laboratories, Institute of Health and Biomedical Innovation, Queensland University of Technology, Brisbane, QLD, Australia; ^2^School of Biomedical Sciences, Queensland University of Technology, Brisbane, QLD, Australia; ^3^Queensland Eye Institute, Brisbane, QLD, Australia; ^4^School of Psychology, University of Queensland, Brisbane, QLD, Australia; ^5^School of Optometry and Vision Science, Queensland University of Technology, Brisbane, QLD, Australia

**Keywords:** pupil, melanopsin, light exposure, depression, MDD = major depressive disorder

## Abstract

**Background:** Melanopsin-expressing intrinsically photosensitive Retinal Ganglion Cells (ipRGCs) signal non-imaging forming effects of environmental light for circadian phoentrainment, the pupil light reflex, and mood regulation. In seasonal affective disorder, ipRGC dysfunction is thought to cause abberant transmission of the external illumination for photoentrainment. It is not known if patients with non-seasonal depression have abnormal melanospin mediated signaling and/or irregular environmental light exposure.

**Methods:** Twenty-one adults who live in a sub-tropical region, including eight patients with non-seasonal depression and thirteen age-matched healthy controls were recruited. The Mini International Neuropsychiatry Interview diagnosed the presence of a major depressive disorder. Light exposure was determined using actigraphy over a 2 week period. The melanopsin mediated post-illumination pupil response (PIPR) and outer retinal inputs to ipRGCs (transient pupil response and maximum pupil constriction amplitude) were measured in response to 1 s, short and long wavelength light with high and low melanopsin excitation.

**Results:** The mean daylight exposure as a function of clock hours and total light exposure duration (mins) to illumination levels commonly recommended for depression therapy were not significantly different between groups. Out of 84 pupil measurements (42 each in the depression and control groups), the melanopsin-mediated PIPR amplitude, transient pupil response, and pupil constriction amplitude were not significantly different between groups.

**Conclusions:** This report provides initial evidence of normal melanopsin function and environmental light exposures in patients with pre-dominately mid and moderate non-seasonal depression in a subtropical location in the southern hemisphere.

## Introduction

The discovery of projections of melanopsin-expressing intrinsically photosensitive Retinal Ganglion Cells (ipRGCs) to brain mood centers (1) has redefined understanding of light-mediated mood regulation (2, 3). These inner retinal cells have major roles in non-image-forming functions including entrainment of the body clock to a ~24 h day-night circadian rhythm (4), regulating the pupil light response (5, 6) as well as in image forming brightness perception, temporal, and color processing in humans (7–9). The post-illumination pupil response (PIPR) (6) is the sustained pupil constriction to high irradiance, short wavelength light that is controlled by the intrinsic melanopsin response from ~1.8 s onwards after light offset (10). During light stimulation, outer retinal inputs to ipRGCs can be assessed with the transient pupil light response (transient PLR) and peak constriction amplitude, with melanopsin contributing to the maintenance of pupil constriction (11, 12).

In seasonal affective disorder (SAD), the melanopsin-mediated PIPR is attenuated (2) whereas daily light exposure and time spent under bright illumination (>1,000 lux) is not different to healthy individuals (13), indicating that impaired light signaling due to ipRGC dysfunction can influence mood in SAD. In contrast, the PIPR amplitudes were normal in a combined cohort of patients with major depressive disorder (MDD) with SAD (*n* = 7) and non-seasonal depressive disorder (*n* = 12) (14). These non-significant differences may reflect the mixed cohort of patients (with and without seasonal depression) and the high variability in the data in the MDD group (14). In MDD patients and healthy controls in the northern hemisphere, the PIPR amplitude is less pronounced in winter compared to the summer months with longer daylight hours (14) and irrespective of season, the transient pupil response to dim light is impaired in MDD, which may reflect dysfunctional outer retinal inputs to ipRGCs (14). The link between light exposure and melanopsin function in patients with MDD, and in particular in non-seasonal depression, has not been examined. This study therefore objectively determined the mean daylight and daily hourly light exposure and duration of exposure to illumination levels recommended for light therapy in depression (15) in a group of patients with non-seasonal depression and healthy participants, and quantified melanopsin function with an optimized pupillometric paradigm that is robust to the presence of subtle melanopsin defects at early stages of retinal disease (16, 17).

## Methods

### Participants

Twenty-one participants aged between 18 and 61 years, including eight patients (38 ± 15 yrs) with non-seasonal depressive disorder (seven female and one male) diagnosed as mild (*n* = 1) moderate (*n* = 5), and severe (*n* = 1) MDD and thirteen age-matched healthy controls (30 ± 7.7 yrs) (6 females, 7 males) were recruited. The recruitment period was classified as summer dominating months (November–March) or winter dominating months (April–August) in Brisbane, Australia, a sub-tropical location in the southern hemisphere. The MDD group without seasonal depression were assessed by a clinical psychologist using the Mini International Neuropsychiatric Interview (MINI) (18), and had at least mild levels of depressive symptoms in the last 2 weeks on the Beck Depression Inventory-II (19) and a negative screen on both, clinician-rated and self-report measures of SAD including the Hamilton Depression Rating Scale-Seasonal Affective Disorder (HDRS-SAD) (20) and the Seasonal Pattern Assessment Questionnaire (SPAQ) (21). The healthy control group was required to have a negative screen on all measures of depressive disorders. All participants had normal visual acuity (>6/6), color vision (Lanthony D-15), normal intraocular pressure and ophthalmoscopy. Participants with history of eye surgery or disease, lens opacification > grade 2 on LOCS III, cognitive impairment and/or intellectual disability and those with circadian disruption (e.g., shift workers or had recently traveled different time zones) were excluded.

### Patient assessment

Each of the 21 participants wore an Actiwatch (Geneactiv, Activinsights, Cambridge shire, UK) for 2 weeks. Actigraphy data were used to calculate the participants' individual light exposure duration to the recommended illuminance of broadband white lights (10,000, 5000, and 2500 lux) used for depression treatment (15). Artifacts were detected and removed in 0.8% of the patients with depression and 4% of the control participants. The time to first light, last light, and the global solar exposure (GSE) in the study location was recorded every day for each participant from the websites of WillyWeather (22) and the Australian Government Bureau of Meteorology (23). The subjective sleep onset latency (time to sleep after going to bed) was derived from the participants' self-reported sleep chart. The mid-sleep time derived from their actigraphy data was computed to compare the circadian phase and chronotype between the MDD and control groups (24).

The pupil light reflex was measured in response to a 1 s stimulus using a custom built, extended Maxwellian view pupillometer according to established laboratory procedures (25, 26) between 10 AM and 5 PM to minimize the effect of circadian variation on PIPR function (27). The left pupil was dilated and the consensual pupil response of the right eye was recorded. The stimuli (repeated twice for each condition) included a short (464 nm) and long wavelength (637 nm) light matched to a corneal irradiance of 15.5 log quanta.cm^−2^.s^−1^. The pupil metrics quantified were the 6 s PIPR, the transient PLR and the maximum pupil constriction (peak PLR) (25).

### Statistical analysis

Statistical data analysis was conducted using IBM SPSS 22 (SPSS, NY: IBM Corp). The MDD and control groups were compared on daily average light exposure, duration spent under light levels ≥ 10,000, ≥ 5,000, and ≥ 2,500 lux and pupil metrics using independent *t*-tests or a Mann-Whitney U test. Hourly light exposure (lux) were log transformed to meet the assumptions for parametric statistical analysis. The relationship between non-seasonal depression, pupil metrics, and light exposure was assessed using Pearson's or Spearman's correlation. Simple linear regression models were used to analyse the presence of linear relationship among the variables.

## Results

Given the known effects of Selective Serotonin Reuptake Inhibitors (SNRIs) on the baseline pupil diameter, pupil constriction amplitude and the PIPR (28), a separate analysis excluding the patients taking SNRIs showed no significant group difference in the baseline pupil diameter (*U* = 33.5, *p* = 0.6), and peak PLR (blue, *U* = 34, *p* = 0.7; red, *U* = 36, *p* = 0.8) and PIPR amplitudes (blue, *U* = 37, *p* = 0.9; red, *U* = 26, *p* = 0.3). There are contentious reports of the effects of Selective Serotonin Reuptake Inhibitors (SSRI), with either no effect (28) or increasing pupil diameter (29); in this study, four patients were taking the drug but there were no significant difference in resting baseline pupil diameter compared to the control group. Similarly, other less commonly used psychotropic drugs did not have a significant effect on PIPR amplitudes (antimanic drug, Mann-Whitney *U* = 38, *p* = 0.9; agomelatine, *U* = 43, *p* = 0.9; MAOI (monoamine oxidase inhibitor), *U* = 38, p = 0.6).

There was no significant difference between the groups for age (*U* = 31.5, *p* = 0.1), the global solar exposure (GSE) (*U* = 33, *p* = 0.2) and the photoperiods [*t*_(19)_ = 2.0, *p* = 0.06]. The mean (± SD) BDI-II scores for MDD and control groups were 25.8 ± 5.7 and 0.5 ± 0.9, respectively (*U* = 0, *p* < 0.0001). The sleep length (*U* = 44, *p* = 0.6) and mid-sleep times [*t*_(19)_ = 0.1, *p* = 0.9] were not significantly different between the groups indicating the groups were comparable on circadian phase and chronotype.

**Figure 1:**
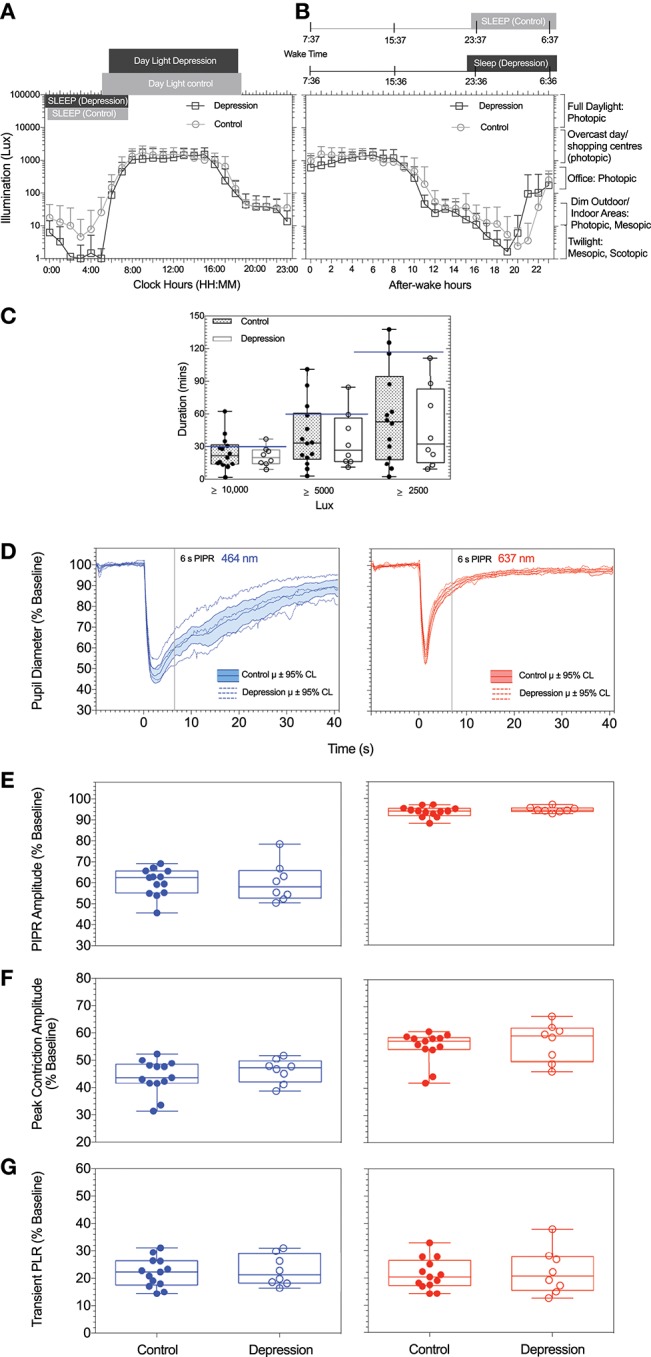
**(A,B)** Average light exposure (illumination, Lux) over a 2-week period as function of **(A)** daily mean (±SD) clock hours and **(B)** circadian hours, with circadian time referenced to individual wake time (defined as zero after-wake time) to mimimize individual differences in light exposure due to differences in participant wake times. **(C)** Daily duration of exposure to illuminance levels recommended for depression light therapy in patients with depression and controls are shown. **(D)** The mean pupil light responses and 95% confidence intervals to the two stimuli (blue, red) for patients with depression and control particpants. For all particpants and both stimulus wavelengths, Box-and-Whisker plots show the median, 25 and 75% quartiles and range of the **(E)** PIPR amplitudes, **(F)** peak constriction amplitude, and **(G)** transient PLR. The blue lines represent the data measured with the short wavelength (blue) light (464 nm); red lines represent the data measured with the long wavelength (red) light (637 nm).

The average light exposure at every clock hour over the 2 week recording period using a 24 h period were not significantly different between the MDD group and healthy controls [*F*_(23, 408)_ = 0.4, *p* = 0.9] (Figures 1A,B). The mean daylight exposure in the MDD group was also not significantly different from controls [*t*_(19)_ = 0.9, *p* = 0.3] (Figure 1B). During the day (8:00–16:00 clock hours), both groups were exposed for at least 8 h after wake time to photopic illumination equivalent to that on an overcast day (~1,000 lux) (Figure 1B). During the evening (16:00–18:00 clock hours), both groups experienced light levels equivalent to an office light exposure (250–500 lux) (Figure 1B). During the night hours, both groups were exposed to illumination level common to the mesopic range and the scotopic ranges (30) and the median nightlight exposure was not significantly different between groups (Mann-Whitney *U* = 50, *p* = 0.9) (Figure 1B).

The light exposure levels were not significantly different between season (summer or winter) or time of day for the two groups [*F*_(23, 408)_ = 0.9, *p* = 0.6]. The duration (mins) of light exposure under illuminations commonly recommended for depression light therapy (≥ 10,000 lux) (15) did not differ between groups (Mann-Whitney *U* = 43, *p* = 0.5), ≥ 5000 lux [*t*_(19)_ = 0.5, *p* = 0.6] and ≥ 2500 lux [*t*_(19)_ = 0.8, *p* = 0.43] (Figure 1C).

Each participant underwent four pupil measurements (2 blue and 2 red) resulting in a total of 84 pupil recordings. Table 1 outlines the mean pupil light reflex data for both groups. Figure 1D shows the mean pupil response (± 95% confidence intervals) to 1 s short wavelength (blue) and long wavelength (red) light stimuli. The baseline pupil diameter was not significantly different between the groups [*t*_(19)_ = 0.7, *p* = 0.5]. The PIPR amplitudes were also not significantly different between groups [*t*_(19)_ = 0.02, *p* = 0.9] (Figure 1E). There was no difference in PIPR amplitudes for participants recruited during the summer and winter dominating months [*t*_(29)_ = 1.4, *p* = 0.2]. Peak constriction and transient PLR amplitudes (Figures 1F,G) were not different between the groups for blue [peak, *t*_(19)_ = 0.9, *p* = 0.4; transient PLR, *t*_(19)_ = 0.4, *p* = 0.7] or red stimuli [peak PLR, Mann-Whitney *U* = 37, *p* = 0.3; transient PLR, *t*_(19)_ = 0.3, *p* = 0.7]. There was no linear relationship between light exposure and PIPR amplitude in the MDD [*F*_(1, 6)_ = 0.6, *R*^2^ = 0.1, *p* = 0.5] or control group [*F*_(1, 11)_ = 2.1, *R*^2^ = 0.2, *p* = 0.2].

**Table 1:** Pupil metrics (mean and 95% CL) for the two participant groups.

**Pupil metrics**	**Short wavelength (464 nm)**		**Long wavelength (637 nm)**	
	**Depression**	**Control**	**Depression**	**Control**
6 s PIPR (%)	60.31 ± 9.2	60.25 ± 6.5	94.7 ± 1.3	93.6 ± 2.4
	95% CL (52.6–68.0)	95% CL (56.4–64.2)	95% CL (93.6–95.8)	95% CL (92.2–95.1)
Peak PLR (%)	46.1 ± 4.4	43.9 ± 6.1	56.9 ± 7.1	55.1 ± 5.7
	95% CL (42.5–49.8)	95% CL (40.2–47.6)	95% CL (51.0–62.8)	95% CL (51.7–58.6)
Transient PLR (%)	23 ± 5.5	22 ± 5.2	22.4 ± 8.2	21.4 ± 5.6
	95% CL (18.3–27.5)	95% CL (19–25.2)	95% CL (15.5–29.3)	95% CL (18–24.7)

*95% CL, 95% confidence limit; PIPR, Post-Illumination Pupil Response; PLR, pupil light response*.

## Discussion

This study provides the initial evidence that there are no significant differences between patients with mainly moderate and mild non-seasonal MDD and healthy controls in either daily and hourly light exposure or the duration spent under bright illuminations commonly recommended for light therapy in MDD. The findings of normal PIPR amplitudes and transient PLR to stimuli with high melanopsin excitation are consistent with a recent study based in the northern hemisphere that investigated 12 MDD patients with non-seasonal depressive disorder (14). That study however, did not record detailed light exposure data in their control group. That a previous study in 15 patients with SAD detected a significant reduction in the melanopsin-mediated PIPR amplitude (2), may indicate different pathomechanisms are involved in SAD, and/or that different ipRGCs subtypes, including those that do not primarily signal to the OPN to regulate the pupil, are differentially affected in the two conditions. M1 ipRGC subtypes have more projections to the SCN than other brain areas (31) which implies that they may have a more prominent role in photoentrainment than in other behavioral functions such as mood. Within M1 subtypes, there is evidence from mouse models that Brn3b-positive M1 ipRGCs project to the OPN shell to control the pupil light reflex and to many other brain areas, whereas Brn3b-negative M1 cells only project to the SCN to drive circadian photoentrainment (32). Based on this, it could be postulated that Brn3b-positive M1 subtypes might be spared in non-seasonal depression, but further evidence is needed in human studies to directly examine this.

We did not find a positive correlation between the PIPR and longer day light hours as previously observed in a study performed in MDD and healthy controls (14). These contrasting findings may be due to the higher light exposure in this geographical area in the southern hemisphere. Importantly, with the melanopsin threshold at ~11.0 quanta.cm^−2^.s^−1^ (33), in our sample light exposure levels were above this threshold throughout their entire wake time in both the summer and winter seasons. Therefore a correlation between the PIPR amplitude and season is not to be expected in study locations closer to the equator.

The small sample of patients with non-seasonal depression is a limitation, and similarly sized to a study evaluating the relationship between non-seasonal MDD and the PIPR in the northern hemisphere (14). We augment existing studies in MDD by providing well defined melanopsin driven pupil responses and initial data on light exposure in a cohort of patients with mainly mild/moderate non-seasonal depression residing in the southern hemisphere. These data can provide a starting point for large-scale, controlled, multi-center studies evaluating the role of the melanopsin pathway in MDD that may lead to targeted, irradiance and wavelength dependent light treatment in MDD in the future.

## Ethics statement

The study followed the Declaration of Helsinki and was approved by the Queensland University of Technology Human research Ethics Committee (Approval Number: 1500000597). Informed written consent was obtained and each participant was offered a $30 gift voucher to compensate for their participation in the study.

## Author contributions

BF and AJZ conceptualized the experiment. BF, AJZ, GO, and LH are responsible for research design, data acquisition, data analysis and interpretation, and manuscript preparation. All authors reviewed the manuscript.

### Conflict of interest statement

The authors declare that the research was conducted in the absence of any commercial or financial relationships that could be construed as a potential conflict of interest.
